# Response: Infant EEG activity as a biomarker for autism: A promising approach or a false promise?

**DOI:** 10.1186/1741-7015-9-60

**Published:** 2011-05-20

**Authors:** William Bosl, Adrienne Tierney, Helen Tager-Flusberg, Charles Nelson

**Affiliations:** 1 Harvard Medical School, Boston, MA, USA; 2 Children's Hospital Boston Informatics Program at Harvard-MIT Division of Health Sciences and Technology, Boston, MA, USA; 3 Harvard Graduate School of Education, Cambridge, MA, USA; 4 Department of Developmental Medicine, Children's Hospital Boston, Boston, MA, USA; 5 Department of Psychology, Boston University, Boston, MA, USA

## 

Please see research article: http://www.biomedcentral.com/1741-7015/9/18 and commentary article: http://www.biomedcentral.com/1741-7015/9/61

The commentary by professors Griffin and Westbury [1] seems to be a response to the widespread media coverage following the recent publication of our article [2]. Most of the media coverage was accurate and carefully emphasized that, while our new approach to measuring early brain development might be promising for detecting risk for disorders such as autism, it has not yet proven that it will yield that desired result and that much more research is still required.

A common problem with the publication of novel medical research on a disease that has a high level of public interest is a tendency to see such work as an imminent breakthrough and/or as having practical clinical implications (such as an early screening tool). This is not entirely negative, as it serves to reinforce the unwritten pact between society and the medical research community: medical researchers are charged with finding cures and relieving suffering. Scientists are required to walk the fine line between pointing out that basic research does indeed have a pragmatic goal in sight, while emphasizing that basic science requires exploration of new ideas, which takes a relatively long time to come to fruition, requiring many small steps along the way.

Our paper, at its core, is about measurable neural correlates of brain development. Our central claim concerns development: "... modified multiscale entropy ... can be used as a biomarker of typical brain development and distinguish typically developing children from a group of infants at high risk for autism spectrum disorder ..." [2].

Many studies suggest that autism is a connectivity disorder [3-6]. Thus, the basic neurophysiological cause for the behaviors that define autism involves a systematic wiring pattern that differs in some consistent manner from those that do not exhibit autistic behaviors. Furthermore, changes in brain developments are known in at least some cases to precede observable changes in behavior (given the limited behavioral repertoire of the young infant, this should come as no surprise). The biophysics of neural networks and results from more general complex network analysis suggest that the time series of electrical potentials produced by the brain will contain information about network structure [7-10]. It is thus reasonable to conjecture that electroencephalography (EEG) signals may contain discernible patterns, reflecting something about the underlying neural networks that precede behavior. In this context, our paper is a first attempt to begin to look at certain EEG signal features that are known to be invariant measures of complex system dynamics to see if they are indeed correlated with behaviors or, in this case, possible endophenotypes.

Figure 4 in our paper shows that there are significant group differences in multiscale entropy (MSE) between the high risk and typically developing infants. It is of particular interest that the trajectories for both groups, in all regions shown, are similar from 6 to 9 and 18 to 24 months. The largest group differences are in changes from 9 to 12 months. This suggests that the developmental trajectories from 9 to 12 months may be of greatest interest for discerning the future outcome of individual infants in these groups, generally supporting the individual results shown in Table 3. Unfortunately, at the time of this study, we did not have enough infants who had measurements at all five ages to enable trajectories for individuals to be analyzed. Nevertheless, the data provide a strong suggestion that distinctly different developmental trajectories are followed from 9 to 12 months in these two groups.

In our discussion, we address the classification accuracy at the different ages. Based on the trajectories in Figure 4, we would expect the greatest differences between individuals in the two groups to be seen at 9 to 12 months. We surmise that these reflect quite different developmental patterns at a time when critical developmental milestones relevant to autism characteristics are reached. After 18 months, the group averages seem to follow similar paths. The majority of infants in the high risk group are expected to develop in such a way as to not be diagnosed with autism spectrum disorder (ASD) We note that some recent studies indicate that about 20% of the high risk siblings will develop autism; this number seems to be borne out by our current data, which now includes over 75 high risk infants. In addition, we can expect, based on other studies of high risk infants, that a further 10% or more of the children will have other early developmental problems, such as delayed language acquisition. Whether there are unique features in the MSE data, and particularly in the growth trajectories, to enable high risk infants that do develop an ASD diagnosis to be distinguished from those who do not, will be determined when we are able to analyze data for all infants after they have graduated from our study and have received a confirmed diagnosis of ASD or no ASD.

We can say for certain that the MSE values between the high risk and typical groups are quite different from 9 to 12 months and after that not so different. It also appears that MSE changes in a discernible fashion during normal early development and that our group of high risk siblings exhibits a different MSE growth trajectory. The MSE features that may differentiate infants who eventually develop an ASD diagnosis cannot be determined in our current study because we had no data at all on which infants will fall into that class. Further interpretation will have to wait until more data are available. Until that time, statements about whether the classification accuracy of our results at different ages support or do not support the possibility that MSE is a developmental indicator that can be used to detect developing autistic tendencies cannot be evaluated.

However, the group differences found in this study are between typically developing infants and those with a genetic predisposition for developing autism spectrum disorder. Although there are many confounds and complexities that may eventually show that MSE trajectories are not useful early ASD biomarkers, these results make it reasonable to ask the question: is EEG complexity an early biomarker for ASD? We hope that other researchers will join in to help answer this question, not to prove or disprove a scientific conjecture, but because it may be enormously helpful to millions of children and their families.

## Abbreviations

ASD: autism spectrum disorder; EEG: electroencephalography; MSE: multiscale entropy;

## Competing interests

The authors declare that they have no competing interests.

## Authors' contributions

WJB wrote the initial draft of this response. CAN, HTF and AT reviewed and edited the draft, contributing comments and insights from their research in cognitive development. All authors read and approved the final manuscript.

## Pre-publication history

The pre-publication history for this paper can be accessed here:

http://www.biomedcentral.com/1741-7015/9/60/prepub
